# Badged up for success: Digital badges enable graduate students to become confident communicators via real-world opportunities and to document their skills for employers

**DOI:** 10.1017/cts.2025.10188

**Published:** 2025-10-29

**Authors:** Kimberly McGhee, Matthew Greseth, Tammy Loucks, Paula Traktman

**Affiliations:** 1 South Carolina Clinical and Translational Research Institute, College of Medicine, Medical University of South Carolina, Charleston, SC, USA; 2 Academic Affairs Faculty, Medical University of South Carolina, Charleston, SC, USA; 3 College of Graduate Studies, Medical University of South Carolina, Charleston, SC, USA

**Keywords:** Science communications, digital badge, microcredential, career development, graduate education

## Abstract

In January 2023, the South Carolina Science Writing Initiative for Trainees (SC-SWIFT), an internship in the College of Graduate Studies at the Medical University of South Carolina, began offering tiered digital badges in science communications. The badges’ purpose was to encourage graduate students and postdoctoral fellows to engage in extracurricular science writing opportunities available through SC-SWIFT and to document acquired communications skills for employers. The badges have been well received, with 18 interns earning the beginner badge in the first two years of the program. In March 2025, SC-SWIFT queried 25 interns who had earned a beginner badge or completed half the requirements for doing so in 2023–2024 to gauge how important they considered the badges to their engagement in science communications and how valuable they would be in a job search. All 14 respondents found the badges important in engaging them in science communications, and 86% either strongly agreed or agreed that digital badges would be an asset when job searching. Eleven of 12 respondents (92%) thought that their confidence in telling their own research story had increased. These initial results suggest that digital badges could be useful tools for documenting science communications skills acquired during extracurricular, experiential learning.

## Introduction

It is critical to prepare graduate students and postdoctoral fellows in biomedical sciences for careers both inside and outside of academia, as the number of life sciences PhDs in the U.S. has quadrupled in the past 50 years with no increase in faculty jobs [1]. This dearth of academic jobs relative to the number of trainees, together with increasingly competitive National Institutes of Health funding, has led many trainees to turn to industry and other science-adjacent areas for employment. In response, graduate programs are placing increased emphasis on transferrable skills, which are not specific to a particular job but instead crucial to success in many types of jobs [2]. These “transferable” skills that nonacademic and academic employers alike have come to expect from applicants with advanced biomedical science degrees [3] are learned not only through traditional coursework but through extracurricular, experiential opportunities [4-7].

Among transferable skills, students believe communications skills to be the most important for their future employability [8]. Science communications skill acquisition and productivity, in particular, predict a greater likelihood of pursuing a research career, whether in or out of academia [9]. These skills are recognized to be important for instilling trust in science and willingness to participate in clinical research, as well as improving the public’s scientific literacy [10].

The importance of science communications skills to effective research has been recognized by leading research organizations. The National Academies of Science, Engineering and Medicine advises that students in the 21st century should “[a]cquire the capacity to communicate, both orally and in written form, the significance and impact of a study or a body of work to all STEM professionals, other sectors that may utilize the results, and the public at large” [11]. The National Center for Advancing Translational Sciences has identified being “a skilled communicator” as one of the seven characteristics of an effective translational scientist [12]. Certainly, the ability to talk science beyond the jargonistic constraints of a specific field is crucial to building the multidisciplinary collaborations needed to advance research breakthroughs from bench to bedside and share those breakthroughs with community members and their providers. To address this need, several Clinical and Translational Science Awards hubs have reported on initiatives to improve science communications skills in trainees [13] and team members, including rubrics [14], workshops [15], and art [16].

Though the past decade has seen a growing acknowledgment of the need for scientists to communicate with broader audiences, most training programs still focus primarily on developing academic writing skills, even though at least half of students will pursue nonacademic careers [17,4-6], and most science communications training provided is limited to electives or workshops, with little opportunity to practice and refine skills [18-20]. The content of science communications training offerings can seem somewhat opaque to employers [21], who don’t know which lay communication techniques students and postdoctoral fellows have been exposed to and which, if any, they have tried their hands at. Yet it has proved challenging to document the acquisition and development of science communications and other transferable skills, particularly when they are gained outside the classroom. Digital badges could be a transparent means of doing so, as they are intended to be shared via social media and provide both learning objectives and metadata that specify how each student achieved those objectives and met badge requirements [22-24].

To encourage students and postdoctoral fellows to take advantage of real-world, extracurricular science communications opportunities available via the College of Graduate Studies (CGS) at the Medical University of South Carolina (MUSC) and to help them highlight these skills for employers, the South Carolina Science Writing Initiative for Trainees (SC-SWIFT) [25], a science communications internship in the CGS, began offering tiered digital badges (beginner, intermediate, advanced) in January 2023. In March 2025, SC-SWIFT queried interns who had completed one or more badges in 2023 or 2024 as well as those who had completed at least half the requirements for the beginner badge to learn more about how they perceived the badges and their value. In particular, we asked whether the availability of digital badges had increased interns’ engagement in science communications opportunities and their confidence in their lay communications skills and whether they saw the badges as assets in their job search. Their feedback, reported here, provided an early snapshot of the usefulness of digital badges in building interest in science communications opportunities and confidence in science communications skills, as well as gauged intern perceptions of the value of digital badges in documenting science communications skills for employers.

## Methods

Established in 2016 for postdoctoral fellows and expanded to graduate students in 2018, SC-SWIFT provides trainees the opportunity to write and publish news stories and releases about recent MUSC research and to write on any science topic for the CGS blog, *CGS Speaks*. The internship is a collaboration between the CGS; the South Carolina Clinical & Translational Research (SCTR) Institute, the clinical and translational science awards hub at MUSC; and MUSC’s Office of Communications and Marketing [25]. Open to all graduate students and postdoctoral trainees, the internship has been of special interest to graduate students selected for the T32 training grant, titled “Cellular, Biochemical and Molecular Sciences Training Program: Developing the skills and expertise needed for a changing biomedical landscape” (CBAMS; initiating principal investigator: Dr Paula Traktman), which was awarded to the CGS in 2019 and renewed in 2024. The program requires trainees to become involved in two of four areas of concentration: science communications, entrepreneurship, science education, and community engagement/advocacy.

The CGS at MUSC enrolled 27 PhD students and 7 MD/PhD students in 2023, and 32 PhD students and 7 MD/PhD students in 2024. During its initial funding cycle (2019–2024), the CBAMS T32 training program supported four CGS students each year, with that number increasing to six with its renewal. Of the 26 CBAMS trainees who have chosen tracks as of November 2024, 19 (73%) have opted for the concentration in science communications, making it the most popular of the four tracks.

**Figure 1.**
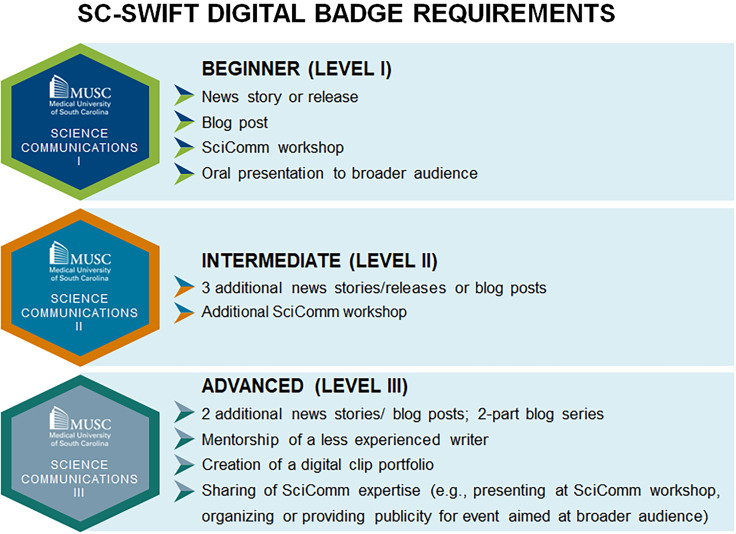
Requirements for the South Carolina Science Writing Initiative for Trainees (SC-SWIFT) tiered badges in science communications.

A long-term CGS goal had been to establish digital badges to document skills gained by students and postdoctoral trainees as they participate in robust CGS offerings in science communications, entrepreneurship, outreach, advocacy and teaching. MUSC established a university-wide approval process for digital badges, and CGS and SC-SWIFT leadership applied to offer beginner, intermediate, and advanced digital badges in science communications, receiving approval in December 2022. To gain approval, SC-SWIFT developed learning objectives (Supplemental Material 1), specified which requirements had to be met to earn each badge (Figure 1), and described how experienced science writers would mentor students and evaluate their work. Publication of written works to the MUSC Catalyst News Center, EurekAlert! or the *CGS Speaks* or other science-related blog is required for completion of the badges. This requirement has not been a barrier, as the science writing mentors work through as many drafts as it takes for interns to arrive at publishable pieces.

The digital badges document both participation in workshops where science communications skills are learned and completion of real-world communications projects where these skills are applied and developed. Students who complete the badges walk away with an online portfolio of published stories documenting their ability to communicate across scientific disciplines and to a variety of audiences, including the public.

To award the badges, SC-SWIFT opted to use Brightspace, the learning management system used by MUSC. The requirements for each badge were loaded in as assignments, enabling trainees to post documentation of completed requirements easily. Because requirements involve experiential learning such as writing news releases/stories for publication and not self-graded quizzes, as are used in other programs that automate the awarding of digital badges, SC-SWIFT leadership opted to award digital badges manually through the “Awards” function in Brightspace once an intern had met all requirements for a given badge. SC-SWIFT leadership checks progress towards badge completion weekly and awards badges as appropriate.

Interns must pursue badges consecutively to ensure they have attained a solid foundation with the beginner badge before moving on to pursue stories more independently and mentor less experienced communicators. Interns are expected to do some stories outside their area of expertise and to try their hand at stories on translational or clinical research as well as basic science and to showcase them in their final online portfolio (Supplementary Material 2).

To inform students and postdoctoral fellows about the initiative, SC-SWIFT leadership announced the availability of digital badges in science communications in the weekly CGS newsletter, emailed current interns about the opportunity, and introduced the badges to the incoming class of biomedical science graduate students via guest lectures in first-year courses and while teaching the Scientific Writing as Persuasion course, required of all PhD students in the summer after their first year. In addition, trainees in the CBAMS T32 Training Program were informed that they would need to earn a beginner badge through SC-SWIFT to fulfill the requirements of the program’s science communications track. SC-SWIFT also expanded its offering of training workshops, required for the beginner and intermediate badge, focusing on writing workshops in the fall and visual rhetoric workshops in the spring.

Since the rollout of digital badges in January 2023, 18 interns have earned at least the beginner digital badge, seven have also completed the intermediate badge, and one has completed all three badges. Among this group, three completed their doctoral training and graduated. Although SC-SWIFT is open to postdoctoral fellows, all badge earners thus far have been either PhD (16 [89%]) or MD/PhD (2 [11%]) students at the time of the receipt of their badge. This is not surprising as most efforts to raise awareness have focused on announcements in first-year classes, and the summer writing class provides students a jumpstart on the beginner badge.

To gauge attitudes towards the recently instituted badges and perception of their value, in March 2025 SC-SWIFT sent a REDCap questionnaire (Supplementary Material 3) to 25 SC-SWIFT interns who had completed a digital badge (15 [60%]) or at least half of the requirements for the beginner digital badge (10 [40%]) and had not graduated from MUSC. Recipients were asked to gauge their level of agreement or disagreement with eight statements about the badges using a 5-point Likert scale (strongly agree, agree, neutral, disagree, strongly disagree). They were also asked to comment on the strengths and limitations of the digital badge program, the importance of science communications, and the value that the badges brought to their job search

## Results

Of the 25 interns surveyed, 14 completed the questionnaire, for a response rate of 56%. Respondents were asked to gauge their level of agreement or disagreement with eight statements about the digital badge program, five of which addressed the importance digital badges played in engaging them in science communications opportunities and building their confidence in their written and oral communications skills, and three of which focused on the badges’ usefulness as assets for the job market.

All 14 respondents either strongly agreed (9 [64%]) or agreed (5 [36%]) that the badges had increased their engagement in science communications opportunities offered by SC-SWIFT. Of the eight badge earners who reacted to the statement “I plan to pursue more than one level of digital badge in science communications,” 7 (88%) either strongly agreed (5 [63%] or agreed (2 [25%]) and 1 (12.5%) remained neutral.

When asked to express their agreement or disagreement with statements about the program increasing confidence in written and oral science communications skills, 75% either strongly agreed (7 [58%]) or agreed (2 [17%]) that their confidence in their written skills had improved, while 3 (25%) remained neutral, and 83% of respondents either strongly agreed (6 [50%]) or agreed (4 [33%]) that their confidence in their oral communications skills had improved, with 2 (17%) remaining neutral (Table 1). The CGS has offered an annual three-minute thesis competition for the past four years, and participation in the event counts toward the digital badge. Survey findings suggest that this opportunity, as well as others (e.g., the Lab to Leaders lay poster competition), has increased student confidence in their oral communications skills. Of the 12 respondents who rated their agreement with a statement that their confidence in their ability to tell their own research story to a variety of audiences had improved, 92% thought that it had, with 8 (67%) strongly agreeing, 3 (25%) agreeing, and 1 (8%) remaining neutral.

**Table 1.** Self-reported motivation and confidence of SC-SWIFT interns who took part in a tiered science communications digital badging practical experience

Survey statements	No.(%) of Respondents		
	Strongly agree	agree	neutral
**Motivation**			
“The availability of digital badges has increased my motivation to engage in science communication opportunities.” (*N* = 14)	9(64)	5(36)	0(0)
“I plan to pursue more than one level of digital badge in science communications.” (*N* = 8)	5(63)	2(25)	1(13)
**Confidence**			
“I am more confident in my written science communication skills.” (*N* = 12)	7(58)	2(17)	3(25)
“I am more confident in my oral science communication skills, including my presentation and interviewing skills.”(*N* = 12)	6(50)	4(33)	2(17)
“I am more confident in being able to tell my own research story to broader audiences.” (*N* = 12)	8(67)	3(25)	1(8)

SC-SWIFT = South Carolina Science Writing Initiative for Trainees.

Of the 14 respondents, 86% either strongly agreed (7 [50%]) or agreed (5 [36%]) that digital badges are an asset in the job search (Table 2). As to sharing the badges via online profiles, 92% of 12 respondents either strongly agreed (8 [67%]) or agreed (3 [25%]) that they had, and 83% either strongly agreed (7 [58%]) or agreed (3 [25%]) that they used the badges to document science communications skills.

**Table 2.** Value of digital badges as markers of professional development in science communications

Survey statements	No.(%) of Respondents (N = 12)			
	Strongly agree	agree	neutral	disagree
“I have shared or plan to share my badge on my online profiles.”	8(67)	3(25)	1(8)	0(0)
“I have used the digital badge(s) and online portfolio of stories to document my science communication skills.”	7(58)	3(25)	1(8)	1(8)
“I consider the digital badges in science communications an asset in my job search.”	7(50)	5(36)	2(14)	0(0)

Program strengths identified by the survey respondents included the opportunity to work with professional writers who gave detailed feedback, to have both didactic and hands-on learning opportunities, to write for a variety of audience groups and to make oral presentations to broader audiences. They also liked that they could pitch their own stories and that the program enabled them to generate published clips to document their science communications skills. Respondents also enjoyed interviewing subject matter experts on campus about their recent research, including those outside their own field, and thought that doing so improved their interviewing skills and ability to discuss science beyond disciplinary boundaries (Table 3). One intern even commented that the program helped him to avoid becoming burned out by his lab research (Table 3).

**Table 3.** SC-SWIFT intern perspectives on the value of the digital badge program

“The science communications badge program has helped me develop the ability to take in complex scientific information, digest it, and effectively communicate it to audiences at multiple levels of scientific literacy. (…) These skills will be essential for me in the future as a physician-scientist, as I aim to bridge cutting-edge scientific research and medical practice.”
“The internship allowed me to think about topics outside of my research at a high level and translate their relevance effectively at the patient and healthcare provider levels, which is a crucial attribute of almost any science-related job one could have after graduating.”
“The science communications internship helped me land my dream job because the skills and confidence I gained are transferable to various attributes that my employers were looking for. Additionally, it is a unique opportunity not many other universities offer, allowing me to stand out as a candidate during my interview process.”
“This internship helped me discover my love for science communication. I did not know about science writing before this program, and now I plan on pursuing science journalism upon graduation.”
‘In times where I was feeling burned out by my laboratory research, I found that working towards the badges and participating in the workshops helped reignite my passion for science and even think about my project in different ways.’

SC-SWIFT = South Carolina Science Writing Initiative for Trainees.

The respondents also identified opportunities for program improvement, with several respondents desiring more science communications workshops, some with external workshop facilitators, as well as more invited speakers with various career paths in science communications. Students also expressed the desire for more audiovisual opportunities, such as making videos and podcasts, a long-held goal of the program that has been hampered by limited funds and access to expertise. Finally, some respondents wanted a more organized way of being notified of potential stories (currently done via email), suggesting a digital document that could be updated as stories were claimed or more stories became available.

Of the 14 interns who responded to the question about career plans, 4 (29%) identified academic research, 1 (7%) industry research, 4 (29%) science or medical communications and 5 (36%) “other” as their chosen career path. We were able to follow up with three of the respondents who chose “other,” and they identified their chosen (or actual, for those who had already landed a job) career path as physician-scientist, venture capitalist, and life sciences strategy consultant.

When asked to comment on how the badges had helped to prepare them for their chosen career, we received extremely positive feedback (Table 3), though one intern thought we should continue to promote digital badges with employers so that they would be more likely to recognize their value. The intern who credited the internship and digital badge program with igniting her interest in science journalism (Table 3) was recently named one of 17 Mass Media Fellows nationwide by the American Association for the Advancement of Science and was embedded as a science writer for the summer at a high-circulation newspaper in North Carolina [26].

## Discussion

Graduate-level science communications programs are still sparse in biomedical science, and most do not provide hands-on experience, such as the opportunity of “doing” science communications by creating real-world pieces [19,27]. Like Longnecker [19], SC-SWIFT sees “learners as apprentices entering a field of activity,” considers “the production of communications resources to be highly appropriate assessments” of their mastery of lay-writing techniques and is confident that these learner-created communications are useful to institutions by helping get the word out about recent high-impact research [28-29]. MUSC faculty have been highly supportive of the internship, believing that the intern-authored news stories and releases are bringing more national attention to their research. SC-SWIFT draws on the expertise of science communicators and researchers on campus and reflects best practices of professional science communicators, as it is run by professional science writers and adheres to the publication conventions of campus publications and national communications outlets. It provides students with real-world writing experiences that enable them to practice and master skills learned in didactic science communications workshops and to document that experiential learning through digital badges.

The tiered digital badges in science communications have provided a structured learning pathway [30] for interns, from mastering the basics of science communications for the beginner badge, to honing their skills with continued practice for the intermediate badge, to achieving greater autonomy and independence in completing writing projects and providing mentorship for less experienced writers for the advanced badge. Our survey results confirm findings by a previous study that digital badges increased students’ interest in acquiring transferable skills [31], in this case by engaging in science communications opportunities offered by SC-SWIFT. The intent of most interns in our survey to pursue additional badges stands witness to both the value interns place in them and their ability to engage students in science communications. In their comments, interns appreciated both the autonomy they are given to choose stories and pursue badges at their own speed and the detailed feedback they are provided, both of which have been shown to increase intrinsic motivation [32]. Interns are encouraged to cover a mix of basic, clinical and community-based research for a final portfolio [33] that spans the translational spectrum and to share their stories via their online profiles, but are also given the autonomy to pursue stories that interest them to keep their interest level high.

Survey results also suggest that participation in SC-SWIFT and pursuit of a digital badge in science communications increased most interns’ confidence in their ability to communicate science to broader audiences in both oral and written formats, with most interns responding that they were more confident in their ability to tell their own research story. Most interns also saw the badges as assets in their job search and considered them worthy of sharing to their online profiles, a noteworthy metric as many badges go unclaimed because students don’t recognize their value [34].

In their comments, interns also noted that pursuing the badges as part of the internship had caused them to step outside their comfort zone, broadened their view of science and taught them to discuss complex ideas outside their field in simple terms and for a variety of audiences. Interns considered these important skills for both academic and nonacademic jobs, as in both they will be required to tailor persuasive messages for a variety of audiences.

## Future steps

More efforts are needed to raise awareness of the digital badges in science communications among postdoctoral fellows. We plan to message this community about the availability of the badges through the MUSC Associate Dean for Postdoctoral Affairs and the MUSC Postdoctoral Fellow Association.

The current study was meant to be an early snapshot of the reception and perceived utility of the new digital badges in science communications. Although the questionnaire we used was standardized in the sense that the same survey was sent to all recipients, it was not informed by a widely recognized instrument for assessing motivation and self-confidence. For longer-term program evaluation, we plan to adopt the Science Communication Training Effectiveness (SCTE) Scale [35], or a modification thereof, which uses Likert scales to assess motivation, self-confidence, self-efficacy, cognition, affect, and behavior. Going forward, we will require interns to complete the instrument when they first express an interest in the internship and digital badge program (as a baseline) and again as they complete each additional digital badge, using these data to continuously improve the internship and the digital badge program.

Only a few studies have looked at how employers value digital badges [6,21] and which skills they would like to see incorporated into the badge requirements. We plan to survey select employers in both academic and nonacademic fields of interest to students (e.g., academic scientists, physician-scientists, industry researchers, consultants, medical liaisons, medical writers) as well as employers of alumni who earned badges to see if they consider the current badges useful barometers of the students’ science communications skills and whether other skills need to be added. Once more badge earners have graduated and begun or completed their job search, we will also query them about how they incorporated mention of the badges into their resumes/CVs and/or online profiles, how useful the online portfolios of writing samples were to their job search, and how frequently employers discussed the badges and portfolios at interviews. We will revise digital badge criteria as needed to ensure that graduates have the communications skills most valued by both academic and nonacademic employers and crucial for a successful career in a scientific or science-adjacent field in the 21st century.

## Conclusion

Our survey of SC-SWIFT science communications interns provides a snapshot from one biomedical science graduate program that digital badges are well received by students, increasing their engagement in science communications initiatives and confidence in their science communication skills while also providing a means to document those skills, even those gained through extracurricular activities, for both academic and nonacademic employers.

## Supporting information

10.1017/cts.2025.10188.sm001 McGhee et al. supplementary material 1 McGhee et al. supplementary material

10.1017/cts.2025.10188.sm002 McGhee et al. supplementary material 2 McGhee et al. supplementary material

10.1017/cts.2025.10188.sm003 McGhee et al. supplementary material 3 McGhee et al. supplementary material

## Supplementary material

The supplementary material for this article can be found at https://doi.org/10.1017/cts.2025.10188.
