# Supplementary material for: Badged up for success: Digital badges enable graduate students to become confident communicators via real-world opportunities and to document their skills for employers
Source: J Clin Transl Sci. 2025 Oct 29;9(1):e254. doi: 10.1017/cts.2025.10188 (PMC12766514; doi:10.1017/cts.2025.10188)
Supplement: McGhee et al. supplementary material 1 — McGhee et al. supplementary material [file S205986612510188Xsup001.docx]

**Supplementary Table. Digital Badge Learning Objectives**

| **Level 1 (Beginner: Mastering the Basics)** |
| --- |
| Communicate science in plain English, avoiding jargon. |
| Under the guidance of a mentor, apply basic lay-communication skills to engage and inform the public about science, in both written and oral form. |
| Create engaging science blog content on topics assigned by an editor. |
| **Level 2 (Intermediate: Honing Your Craft)** |
| Demonstrate knowledge of an expanded repertoire of lay-communication skills (e.g., tailoring headline and lead paragraph to interest the target audience, use of analogies to help ground the unfamiliar in the familiar, use of storytelling and satisfying story arcs to engage broader audience in science). |
| Confidently apply a variety of lay-writing techniques to engage and inform the public about scientific research across the translational spectrum (e.g., basic, clinical, entrepreneurial). |
| Demonstrate the ability to identify scientific topics of relevance to the public and craft engaging blog posts about them for that audience. |
| **Level 3 (Advanced: Achieving Independence, Mentoring)** |
| Demonstrate proficiency at communicating basic, translational and clinical research and biomedical entrepreneurial activities by completing an e-portfolio of published communications pieces. |
| Promote best practices in science communications by contributing to science communications training workshops, helping to organize lay-friendly posters or three-minute thesis presentations or publicizing other scientific events geared toward broader audiences. |
| Mentor beginning writers one-on-one as they complete communications intended for the public. |
| Demonstrate editorial judgement and leadership by identifying scientific topics of relevance to the public and recruiting and mentoring students to write blog posts about them. |
