# Supplementary figures and images for: Badged up for success: Digital badges enable graduate students to become confident communicators via real-world opportunities and to document their skills for employers
Source: J Clin Transl Sci. 2025 Oct 29;9(1):e254. doi: 10.1017/cts.2025.10188 (PMC12766514; doi:10.1017/cts.2025.10188)

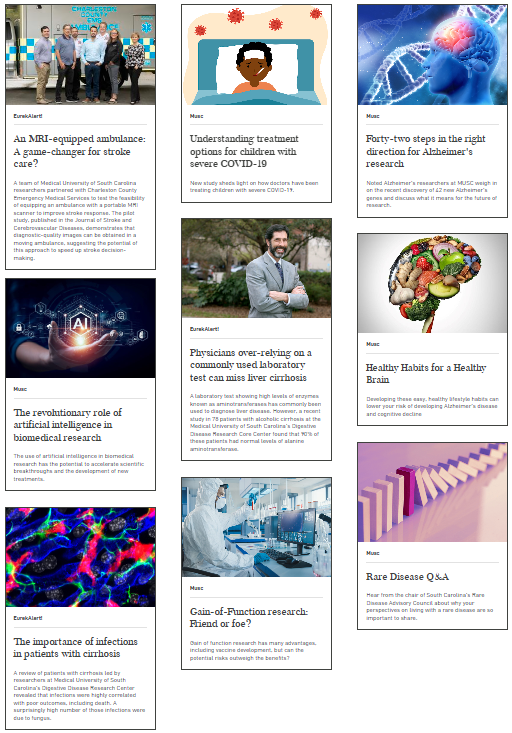

Supplement: McGhee et al. supplementary material 2 — McGhee et al. supplementary material [file S205986612510188Xsup002.tiff]
