# Supplementary material for: Badged up for success: Digital badges enable graduate students to become confident communicators via real-world opportunities and to document their skills for employers
Source: J Clin Transl Sci. 2025 Oct 29;9(1):e254. doi: 10.1017/cts.2025.10188 (PMC12766514; doi:10.1017/cts.2025.10188)
Supplement: McGhee et al. supplementary material 3 — McGhee et al. supplementary material [file S205986612510188Xsup003.pdf]

Science Communications Digital Badge Survey

Hello,

Please take a moment to complete the below survey about the digital badge in science communications. Data collected will be used for an academic paper on the digital badge efforts, and constructive criticism will be helpful in improving the initiative. No identifying data (e.g., name) will be included in the paper. It will only be used for data analysis.

Thank you!

Kimberly McGhee

What is your educational status?

- ◯ Ph.D. student
- ◯ M.D., Ph.D. student
- ◯ M.S. student
- ◯ Postdoctoral fellow

What is your area of specialty?

____

What are your current career plans?

- ◯ Academic researcher
- ◯ Industry researcher
- ◯ Science/Medical Communications or Publications
- ◯ Medical Science Liaison
- ◯ Non-Profit researcher or communicator
- ◯ Other

How many digital badges have you completed?

- ◯ < 1
- ◯ 1
- ◯ >1

Please indicate your level of agreement with the following statements about the digital badge in science communications.

	Strongly agree	Agree	Neutral	Disagree	Strongly disagree
The availability of digital badges has increased my motivation to engage in science communication opportunities.	◯	◯	◯	◯	◯
I plan to pursue more than one level of digital badge in science communications.	◯	◯	◯	◯	◯
I am more confident in my written science communication skills after having earned one or more digital badges.	◯	◯	◯	◯	◯

I am more confident in my oral science communication skills, including my presentation and interviewing skills, after having earned one or more badges.	○	○	○	○	○
I am more confident in being able to tell my own research story to broader audiences after earning one or more digital badges.	○	○	○	○	○
I have shared or plan to share my badge on my online profiles.	○	○	○	○	○
I have used the digital badge (s) and online portfolio of stories to document my science communication skills.	○	○	○	○	○
I consider the digital badges in science communications an asset in my job search.	○	○	○	○	○
Do you intend to complete the beginner's badge in science comnunications?	○	○	○	○	○

If you have encountered obstacles in completing your beginner's badge, what are they?

____

In your view, how will the science communications badge(s) (and internship) help to prepare you for your chosen career?

____

What are the strengths of the current digital badge opportunities in science communications?

____

What suggestions do you have for improving the digital badge initiative in science communications?

____

Last name

____

First name

____
